# Tibetan medicine pain relieving plaster for treatment of knee osteoarthritis: protocol for a multi-center, randomized, and positive drug controlled trial

**DOI:** 10.3389/fmed.2025.1597183

**Published:** 2025-05-15

**Authors:** Yi Yuan, Ying-jie Wang, Ai Guo, Wei-guo Wang, Wei Wang, Pei-lai Liu, Zhi-xiu Shen, Jian-rong Xie, Kai-lie Liu, Min Zhao, Jing-ling Zuo, Kai Liu, Ze-yang Shi, Zhao-lan Liu, Xi-sheng Weng, Jian-ping Liu

**Affiliations:** ^1^Centre for Evidence-Based Chinese Medicine, Beijing University of Chinese Medicine, Beijing, China; ^2^Department of Orthopedic Surgery, State Key Laboratory of Complex Severe and Rare Diseases, Peking Union Medical College Hospital, Chinese Academy of Medical Science and Peking Union Medical College, Beijing, China; ^3^Beijing Friendship Hospital, Capital Medical University, Beijing, China; ^4^China-Japan Friendship Hospital, Beijing, China; ^5^Peking Union Medical College Hospital, Chinese Academy of Medical Science and Peking Union Medical College, Beijing, China; ^6^Qilu Hospital of Shandong University, Jinan, Shandong, China; ^7^Tibet Cheezheng Tibetan Medicine Co., Ltd., Tibet, China

**Keywords:** knee osteoarthritis, pain relieving plaster, flurbiprofen cataplasms, randomized controlled trial, non-inferiority design, trial protocol

## Abstract

**Objective:**

This trial aims to evaluate the effectiveness and safety of a Tibetan medicine pain relieving plaster (PRP) in knee osteoarthritis (KOA) management, generating evidence-based support for clinical application.

**Method:**

This multi-center, randomized, positive drug-controlled, non-inferiority trial will evaluate the effectiveness and safety of the PRP in symptomatic participants with KOA. The trial will enroll 440 participants, allocated in a 1:1 ratio to the PRP group and flurbiprofen cataplasms group. All participants will undergo a 7-day treatment period followed by 14-day post-treatment monitoring. We will assess pain severity by the pain dimension score of the Western Ontario and McMaster Universities Osteoarthritis Index (WOMAC) scale, and evaluate knee joint function using the stiffness and joint function dimension scores of the WOMAC scale and knee joint range of motion. We will assess joint swelling by measuring knee circumference. The quality of life will be evaluated using EQ-5D-5L scale, with utility scores calculated based on utility values and changes in the frequency of each level across dimensions. For safety assessment, we will perform blood laboratory tests, detailed recording of adverse events, and assessment of the severity of local skin reactions to the plaster based on a four-point Likert scale. In addition, we will record information related to plaster use, compliance, and the use of rescue therapy.

**Discussion:**

This methodologically robust randomized controlled trial will comprehensively characterize PRP’s therapeutic potential in KOA management, specifically examining its impacts on core clinical manifestations (pain, mobility restriction, joint swelling) and patient-reported outcomes. The findings will inform evidence-based utilization of this approved Tibetan medicinal formulation in real-world clinical practice.

## Introduction

Knee osteoarthritis (KOA), a chronic degenerative joint disorder, is characterized by progressive articular cartilage degeneration, subchondral bone lesions, and synovial inflammatory reactions. Clinical manifestations primarily include knee pain, swelling, joint deformity, and functional impairment (1). Epidemiological study indicates that 48.5% of middle-aged and elderly KOA patients experience pain as their main symptom (2). Global health data from 2019 indicates approximately 364.6 million KOA cases worldwide, representing 4.9% of the global population (3). According to the Global Burden of Disease database in 2019, the age-standardized prevalence rate has increased by 7.5% since 1990, while the incidence rates rose by 6.2% (4).

Symptom onset typically occurs between 50–59 years, with disability resulting from KOA mainly affecting individuals aged 75 to 84 (4). Currently, the onset of KOA is trending younger, cases of KOA have already been reported in the 30–34 age group, and the number of individuals with KOA in the 40–50 age group is no longer negligible (4). The incidence is higher in women than in men, which may be related to factors such as overweight, obesity, hormone levels, and occupation (4). Comparative analyses demonstrate significantly higher incidence rates for KOA than osteoarthritis at other sites (e.g., hip, hand), with marked geographic variations (5). In the United States, annual per-patient expenditures for osteoarthritis management average approximately 3,000 USD per-person (6), with medical expenditures rising from 10.3 billions USD in 2005 to 185.5 billions USD in 2007 (7). Evidently, KOA imposes a substantial social and economic burden on healthcare systems worldwide (4, 8).

Current clinical practice prioritizes nonsurgical therapies as the first-line treatment for mild to moderate KOA, effectively reducing pain and enhancing functional capacity. For acute symptoms, the Italian Orthopedic and Traumatology Society (SIOT) guidelines (9) for the nonsurgical treatment of KOA recommend oral administration of acetaminophen or topical nonsteroidal anti-inflammatory drugs (NSAIDs). For persistent or refractory symptoms, the SIOT guidelines suggest oral NSAIDs or the use of higher-level analgesic medications (9). Intra-articular injections of corticosteroids and hyaluronic acid are both recommended in the guidelines as first-line treatments for KOA. However, short-term use of corticosteroids (within 2–4 weeks) can effectively relieve pain, whereas long-term use may pose certain risks (9). Intra-articular injections of hyaluronic acid have demonstrated long-term safety, but there is currently limited evidence regarding their efficacy (9). Similarly, oral NSAIDs, topical NSAIDs, and intra-articular corticosteroid injections are also strongly recommended in the American College of Rheumatology (ACR) guidelines (1) for the treatment of KOA.

However, current pharmacological strategies remain predominantly symptomatic, lacking disease-modifying properties (8). Although the guidelines all recommend the use of NSAIDs for the management of KOA, their adverse effects (such as gastrointestinal reactions, cardiovascular risks, and renal dysfunction) cannot be ignored (10–12). Therefore, the guidelines do not recommend NSAIDs for patients with gastrointestinal or cardiovascular diseases, and suggest other patients use only for short-term treatment (13). Interestingly, some studies have reported that NSAIDs may have the analgesia ceiling, meaning that once the medication reaches a certain dosage, it will no longer provide additional pain relief and may instead lead to side effects (14). Longitudinal cohort data (13) reveal long-term use of NSAIDs in KOA patients may actually worsen pain, disability, and stiffness symptoms. Although there was no significant difference in joint structural deterioration compared to KOA patients who did not use NSAIDs long-term, the worsening of symptoms may prompt them to undergo total knee replacement surgery. As the number of emerging therapies continues to grow, exploring and validating a KOA treatment that is both effective and safe holds significant clinical value.

Pain Relieving Plaster (PRP) is a wet compress patch composed of Tibetan herbs such as Duyiwei (Lamiophlomis rotata (Benth.) Kudo) and Jianghuang (*Curcuma longa* L.). In China, it is used for the treatment of various acute and chronic musculoskeletal system diseases, including KOA, acute or chronic sprains, and contusions. Produced by Gansu Cheezheng Tibetan Medicine (Lanzhou, China), PRP has been approved by the National Medical Products Administration of China for marketing (Z54020113) in 1996. Results from relevant animal experiments demonstrate that PRP exhibits anti-inflammatory effects (15), improves blood circulation (16), provides multi-target analgesia (17), and promotes tissue damage repair (18). A systematic review (19) involving 12 randomized controlled trials with a total of 1,197 KOA patients revealed that PRP may have benefits in treating KOA. However, the reliability of these conclusions is reduced due to the low methodological quality of the original studies. This trial aims to evaluate the effectiveness and safety of PRP in KOA patients, methodologically robust multicenter randomized controlled trials are required to systematically assess therapeutic effects on pain intensity, joint mobility limitations, and joint swelling, while evaluating quality of life improvements in KOA patients.

## Methods

### Ethical approval

This trial was approved by the ethics review committee of the Chinese Academy of Medical Sciences and Peking Union Medical College Hospital (I-24PJ1637, Beijing, China), and obtained ethical approval from each sub-center. All participants will sign a written informed consent form prior to enrollment. The development and reporting of the trial protocol adhere to the Standard Protocol Items: Recommendations for Interventional Trials (SPIRIT) checklist (20) and the Consolidated Standards of Reporting Trials (CONSORT) 2010 Statement (21). The trial was registered in ISRCTN registry (ISRCTN10047986). Detailed registration information is available at: https://doi.org/10.1186/ISRCTN10047986.

### Trial design

**Figure 1:**
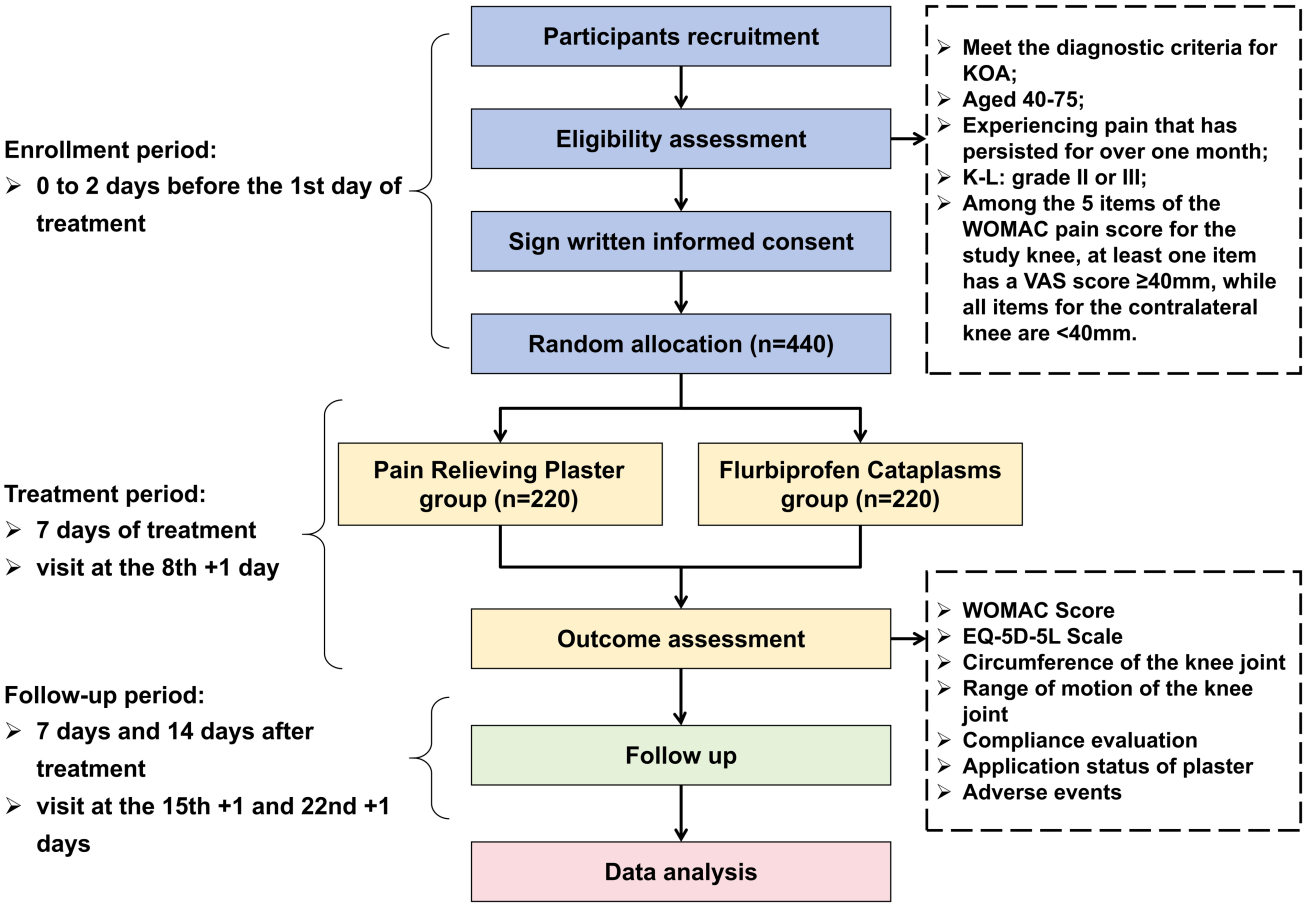
Flowchart of the trial design. KOA, Knee Osteoarthritis; K-L, Kellgren-Lawrence grading scale; WOMAC, Western Ontario and McMaster Universities Osteoarthritis Index; VAS, visual analogue scale; EQ-5D-5L, European Quality of Life 5 Dimensions 5 Levels.

This trial employs a multi-center (7 centers), randomized, parallel-group, non-inferiority design. Eligible participants, after signing written informed consent, will be randomly assigned in an equal ratio to either the PRP group or the flurbiprofen cataplasms group. Enrolled participants will receive medication for a 7-day treatment duration, followed by a 2-week follow-up. The flowchart of the trial design is presented in Figure 1.

### Patient and public involvement

No participants or the public is involved in the design and conduction process of this trial.

### Study setting

This trial will recruit outpatients or inpatients from seven centers in China. Details are provided in Table 1. Patient recruitment will commence on October 1, 2024.

**Table 1:** Study setting details of trial.

No. of center	Hospitals	Location	Role of center	Anticipated participants
1	Peking Union Medical College Hospital, Chinese Academy of Medical Sciences	Beijing	Principle investigator	80
2	Beijing Friendship Hospital Affiliated to Capital Medical University	Beijing	Collaborator	60
3	China-Japan Friendship Hospital	Beijing	Collaborator	60
4	Qilu Hospital of Shandong University	Jinan, Shandong	Collaborator	60
5	Shu Guang Hospital Affiliated to Shanghai University of Traditional Chinese Medicine	Shanghai	Collaborator	60
6	The Second Hospital of Lanzhou University	Lanzhou, Gansu	Collaborator	60
7	Liuzhou People’s Hospital	Liuzhou, Guangxi	Collaborator	60

### Criteria

#### Diagnostic criteria

This trial refers to the diagnostic criteria for KOA established by the ACR in 1995 (22). A diagnosis of KOA can be made if criteria 1, 2, and any one of criteria 3, 4, or 5 are met: (1) knee pain; (2) radiographic osteophytes; (3) age > 50 years; (4) morning stiffness ≤ 30 min; (5) crepitus during movement.

#### Inclusion criteria

Considering the younger age trend of participants with KOA, and to facilitate recruitment and observation of drug effectiveness across different age groups, we have broadened the age range for participants inclusion criteria to 40 to 75 years old.

Eligible participants should meet the following criteria: (1) meet the diagnostic criteria for KOA (either unilateral or bilateral); (2) age between 40 and 75 years; (3) recurrent knee pain for more than one month; (4) Kellgren-Lawrence (K-L) grading scale of II or III; (5) among the five items of the Western Ontario and McMaster Universities Osteoarthritis Index (WOMAC) pain score for the suffered knee, at least one item must have a visual analog scale (VAS) score ≥ 40 mm (0–100 mm), and all pain score items for the contralateral knee must be < 40 mm; (6) willingness to participate in the trial and signing of the informed consent form.

#### Exclusion criteria

Participants meeting any one of the following criteria are not eligible for inclusion in this trial: (1) participants with obvious surgical indications who are not suitable for conservative treatment; (2) participants with other inflammatory painful conditions of the knee, such as rheumatoid arthritis, psoriatic arthritis, gout, pigmented villonodular synovitis, septic arthritis, or tuberculous arthritis. Participants with osteoarthritis caused by osteomyelitis, bone tumors, or bone tuberculosis. Or participants who have undergone joint replacement surgery; (3) participants allergic to the main components of the tested drugs or those with a history of skin allergy to topical medications; (4) participants with skin diseases at or around the knee; (5) participants with liver function abnormalities, i.e., ALT (alanine aminotransferase) or AST (aspartate aminotransferase) levels exceeding 2 times the upper limit of normal, or renal function abnormalities, i.e., Scr (serum creatinine) levels exceeding 1.5 times the upper limit of normal, or participants with severe primary diseases deemed by the investigator as unsuitable for enrollment, including malignant tumors, infectious diseases, and mental illnesses; (6) participants who have taken oral non-steroidal anti-inflammatory drugs within 1 week before screening, oral hormones within 1 month, received intra-articular injections within 3 months, undergone joint surgery within 1 year, or had confirmed knee joint injuries or open wounds within 6 months; (7) Women with positive blood pregnancy tests during screening, as well as pregnant or lactating women; (8) participants deemed by the investigator as having a low likelihood of enrollment or whose enrollment would be complicated by other factors, such as frequently changing work environments or frequent business trips, making them prone to loss to follow-up; (9) participants using traditional Chinese medicine therapies for KOA, such as infrared radiation, cupping, acupuncture, traditional Chinese medicines, etc., or currently participating in other clinical studies.

### Study discontinuation

Participants may withdraw from the trial at any time for any reason, and the specific reason will be recorded in the case report form (CRF). If a patient is erroneously enrolled in the trial due to inappropriate inclusion, the collected data will not be analyzed. If a patient withdraws their informed consent, data collection will cease, but previously collected data may still be used for analysis. Participants may also be withdrawn from the trial if they become pregnant or severely violate the trial protocol. If the investigator deems it necessary to stop the trial for safety reasons or in the best interest of the patient, they will also withdraw the patient. Following withdrawal from the trial, participants will receive other standard treatment options.

### Sample size calculation

The study is a non-inferiority design, with the pain dimension score in the WOMAC score as the primary outcome measure. The sample size for the non-inferiority design trial was calculated using PASS 11 software (23).^1^ According to previous studies (24, 25), the mean pain dimension score in the WOMAC scale after treatment was 6.01 points for the PRP group (with a 4-point scale, totaling 20 points) and 5.46 points for the positive control flurbiprofen Cataplasms group (with a 4-point scale, totaling 20 points). Relevant indicators were set in the PASS software, where *α* represents the probability of making a type I error, with a value of 0.025 (one-sided test). *β* represents the probability of making a type II error, with a value of 0.1 (or the power (1-β) is 0.9). The ratio of the sample size between the experimental group and the control group is 1:1. ∆ is the non-inferiority margin, based on a previous literature review (26), is set at 8 mm (on a 100-point scale). Considering a 20% dropout rate, a maximum of 220 participants are needed in each group, resulting in a total of 440 participants required for the final analysis.

### Randomization, allocation concealment, and blinding

A randomized controlled trial will be conducted using the central block randomization method (with 10 random numbers as a block to ensure balance between groups). Eligible participants will be randomly assigned to one of the two groups, with 220 cases in each group. The SAS software will be used to generate a sequence of random numbers and their corresponding central coding assignments (to specify the range of treatment codes assigned to each clinical center). Within each center, participants will be assigned a number based on the chronological order of their entry into the study. Allocation concealment will be achieved using sequentially coded, sealed, opaque envelopes. Personnel unrelated to the trial will be responsible for allocating and packaging the study medications. The trial medications will be stored in the pharmacy of the participating hospital. Researchers from the participating hospitals will recruit participants, and after signing the informed consent form from the participants, personnel unrelated to the trial will distribute the medications with corresponding serial numbers to the respective participants.

The process of randomization and allocation concealment will be conducted by independent researchers from the Center for Evidence-Based Chinese Medicine, Beijing University of Chinese Medicine (Beijing, China). Prior to patient recruitment, researchers from the participating hospitals will receive standardized training on the trial procedures and evaluation methods to ensure consistency. Data managers and statisticians will remain blinded to the treatment assignments of the participants. This trial will not be blinded for doctors, participants, or outcome assessors due to different interventions.

### Intervention

If a participant is included with bilateral KOA, the side with the severe pain score will be selected as the study joint, and only the selected knee joint will receive medication, while the contralateral knee joint will not be treated. The dosage and administration of both drug groups strictly follow the specifications in their respective package inserts.

### Experimental group

Participants in the experimental group will use PRP during the treatment period. One plaster will be applied each morning, and each plaster will be worn for 8 h. The treatment cycle is 1 week. The application site will be selected as the point of most significant pain around the patient’s knee joint (Ashi point) or the Xiyan point (EX-LE5, anatomical location: located on the extension surface of the knee joint, in the depression on both sides of the patellar ligament; the medial one is called the inner Xiyan point, and the lateral one is called the outer Xiyan point). Remove the plastic film, evenly apply the wetting agent from the small pouch onto the surface of the medicine pad, and after wetting, directly apply it to the selected application site, pressing down the surrounding adhesive tape to secure it.

### Control group

Participants in the control group will use flurbiprofen cataplasms during the treatment period. One patch will be applied each morning and before bedtime, with each patch worn for 8 h. The treatment cycle is 1 week. The method for selecting the application site for the control group is the same as that for the experimental group. Remove the plastic film and apply the patch directly to the selected application site.

### Rescue therapy

During the one-week treatment period, if a patient has a score of 70 mm or above in any of the five pain-related items on the WOMAC scale for their knee joint and requests additional pain medication, non-steroidal anti-inflammatory drugs (acetaminophen tablets) can be used as rescue therapy. Once the score decreases below 70 mm and the pain is alleviated, the rescue therapy will be discontinued.

During the one-week treatment period, if a patient experiences mild skin irritation (such as redness, itching, burning sensation, etc.) at the application site, the medication should be discontinued. Symptoms of mild skin irritation may resolve spontaneously after stopping the medication. Once the skin irritation subsides, medication can be resumed, and it is recommended to shorten the application time as appropriate. If symptoms persist or worsen, the medication should be discontinued immediately, and the patient should seek dermatological consultation promptly.

During the two-week follow-up period, if a participant experiences pain in their knee joint and requests additional pain medication, acetaminophen tablets can be used as rescue therapy.

The researcher can determine the rescue treatment and dosage based on the patient’s and clinical situation, and should record the use of rescue therapy in detail in the CRF form. Participants are not allowed to use infrared therapy, cupping, acupuncture, Chinese herbal medicines, or other topical plaster preparations during the treatment and follow-up periods.

### Outcome measures

The time points for measuring each outcome indicator are shown in Table 2. Baseline data will be collected before participants receive their first treatment, including:

1. Demographic characteristics: ID, age, gender, contact information, ethnicity, occupation, height, weight, marital status, smoking status, alcohol consumption.
2. Disease status: KOA-related onset and treatment information, presence of other chronic diseases.
3. Biochemical and clinical measurements: general physical examination, blood routine, urine routine, blood pregnancy test for women of childbearing age, liver function (including: alanine aminotransferase (ALT), aspartate aminotransferase (AST), alkaline phosphatase (ALP), total bilirubin (TBIL), *γ*-glutamyl transpeptidase (γ-GT)) and kidney function [including: serum creatinine (Scr)], X-ray of knee joint (anterior and lateral positions).
4. Traditional Chinese medicine syndrome differentiation: The doctor will inquire about the patient’s symptoms based on a standardized interview form, and then an experienced TCM practitioner (associate senior professional title or a Chinese medicine practitioner with over ten years of experience in Chinese medical practice) will uniformly conduct TCM syndrome differentiation for the patient.
5. Central Sensitization Inventory scale (CSI-9) (27), expectancy scale for treatment effects and side effects of treatment.
6. Scores on the WOMAC scale (pain, stiffness, and joint function dimensions), EQ-5D-5L score, circumference of the knee joint, range of motion of the knee joint, and concomitant medications (medications taken by the patient for their own chronic conditions).

**Table 2:** Time points and cycles for measuring the outcome indicators.

Assessments and procedures	Enrollment period	Treatment period		Follow-up period			
Time point and cycle	Visit 1 (0–2 days before treatment)	Day 1–7	Visit 2 (8th + 1 day)	Day 8–14	Visit 3 (15th + 1 day)	Day 15–21	Visit 4 (22nd + 1 day)
Data collection at baseline							
Informed consent form	×						
Inclusion and exclusion criteria	×						
Randomization and allocation	×						
Demographic characteristics	×						
Disease situation (including KOA and other chronic diseases)	×						
Traditional Chinese medicine syndrome differentiation	×						
Central Sensitization Inventory (CSI-9)	×						
Expectancy for treatment effects	×						
Expectancy for side effects of treatment	×						
Biochemical and clinical measurements							
Physical examination	×						
Blood routine	×		×				
Urine analysis	×		×				
Blood pregnancy test (only for women of childbearing age)	×						
Liver function (ALT, AST, ALP, TBIL, γ-GT)	×		×				
Renal function (Scr)	×		×				
X-ray of knee joint (anterior and lateral positions)	×						
Effectiveness evaluation							
WOMAC score (pain dimension)	×	Daily recording by participants	×	Daily recording by participants	×	Record once by the participants	×
WOMAC score (stiffness and joint functional dimensions)	×		×		×		×
EQ-5D-5L scale	×		×		×		×
Circumference of the knee joint	×		×		×		×
Range of motion of the knee joint	×		×		×		×
Safety evaluation							
Adverse events		Daily recording by participants	×	Daily recording by participants	×	Record once by the participants	×
Severity of local skin reaction after application		Daily recording by participants	×				
Other evaluation							
Compliance			×		×		×
Drug distribution/retrieval records	×		×				
Combination therapy (used to treat diseases other than KOA)	×	Daily recording by participants	×	Daily recording by participants	×	Record once by the participants	×
Rescue therapy		Daily recording by participants	×	Daily recording by participants	×	Record once by the participants	×
Application duration		Daily recording by participants	×				
Number of plasters applied		Daily recording by participants	×				
The situation of plaster falling off		Daily recording by participants	×				
Medication experience		Daily recording by participants	×				
Use other treatment methods for KOA		Daily recording by participants	×	Daily recording by participants	×	Record once by the participants	×
Engage in vigorous exercise		Daily recording by participants	×	Daily recording by participants	×	Record once by the participants	×

ALT, alanine aminotransferase; ALP, alkaline phosphatase; AST, aspartate aminotransferase; EQ-5D-5L, European Quality of Life 5 Dimensions 5 Levels; KOA, knee osteoarthritis; Scr, serum creatinine; TBIL, total bilirubin; WOMAC, Western Ontario and McMaster Universities Osteoarthritis Index; γ-GT, γ-glutamyl transpeptidase.

In addition, we will provide daily record cards to participants participating in the trial. During the one-week treatment period, the first week of follow-up, and the fourth day of the second week of follow-up, participants will be asked to fill in the daily record cards with scores for the five pain-related items on the WOMAC scale, patch application status (only required during the treatment period), concomitant medications, rescue therapy, and adverse events.

### Primary outcomes

The primary outcome measure is the change in pain dimension scores of the WOMAC scale one week after medication compared to baseline. The VAS application version of the WOMAC scale will be used, with all items scored on a 100 mm VAS scale ranging from 0 mm (no pain) to 100 mm (extreme pain), corresponding to scores from 0 to 100. The pain dimension of the WOMAC scale includes five items: pain when walking on a flat surface, pain when going up or down stairs, pain that affects sleep at night, pain when sitting or lying down, and pain when standing up straight. The pain dimension scores of the WOMAC scale will be measured face-to-face at baseline and at weeks 1, 2, and 3. During the one-week treatment period, the first week of follow-up, and the fourth day of the second week of follow-up, participants will be asked to record their assessments of the previous day’s five items on a daily record card every morning.

### Secondary outcomes

The secondary outcomes mainly include measurements and evaluations of participants’ joint functional activity, joint swelling, and quality of life. Joint functional activity will be assessed using the stiffness and joint function dimension scores of the WOMAC scale. This outcome measure will be self-assessed by participants using the VAS application version of the WOMAC scale, with all items scored on a 100 mm VAS scale ranging from 0 mm (no stiffness or abnormal functional activity) to 100 mm (extreme stiffness or abnormal functional activity), corresponding to scores from 0 to 100. In addition, a wearable joint range of motion (ROM) measurement device (Qindong NEO-SMART, version: QD-1) will be used by the doctor to measure the ROM of the participants’ knee joints. Participants will lie in a supine position on the examination bed and move their knee joints to the maximum extent possible using a heel slide method. The doctor will record the readings from the device. In addition to measuring the ROM of the study knee joint, the ROM of the contralateral knee joint will also be measured as a reference.

The circumference of the knee joint is an indicator used to assess the degree of knee swelling in participants. It is measured using a manual measurement method, as follows:

1. Instruct the patient to lie flat with both legs straight and identify the position of the patella.
2. Mark the positions of the center, upper edge, and lower edge of the patella.
3. Use a tape to measure the circumference around the center, upper edge, and lower edge of the patella. The tape measure should be placed close to the skin with suitable tension.
4. Measure the circumference of the contralateral knee joint using the same method as a reference.

The EQ-5D-5L scale will be used in this trial to assess the quality of life. It includes the following five dimensions: mobility, self-care, usual activities, pain/discomfort, and anxiety/depression. Each dimension has five levels, ranging from no difficulties to extreme difficulties/unable to perform.

All secondary outcomes will be measured face-to-face at baseline and at weeks 1, 2, and 3.

### Safety assessment

Safety assessment encompass blood laboratory tests, adverse events, and the severity of local skin reactions after application. In this trial, blood routine, urine routine, liver function tests (including ALT, AST, ALP, TBIL, *γ*-GT), and renal function tests (including Scr) will be conducted at baseline and during the follow-up visit one week after medication administration. Changes in blood laboratory test results before and after treatment will be compared. Adverse events occurring in both groups during the one-week treatment period and the two-week follow-up period will be recorded, and differences in adverse event rates between the two groups will be compared.

Furthermore, a Likert-type four-point scale (ranging from none to severe) will be used to assess the severity of local skin reactions after application, including subjective symptoms (itching, pain, burning sensation) and skin reaction evaluations (erythema, papules, edema, blisters, exudation, erosion, exudation and ulceration, desquamation, etc.). The severity of local skin reactions after application will be measured face-to-face during the first week of follow-up. Participants will be asked to record any adverse effect on a daily record card every morning during the one-week treatment, reflecting the previous day’s observations.

### Other assessment

We will evaluate patient compliance using two methods: the medication counting method and the follow-up rate method. The medication counting method involves calculating the number of remaining medications when collected after the first week of treatment, comparing it to the actual number of medications dispensed. Participants are considered to have good medication adherence if they have completed 80% or more of the prescribed treatment; otherwise, they are considered to have poor medication adherence. The follow-up rate method assesses compliance based on the number of follow-up visits attended by the participants. This trial includes three follow-up visits, and participants are considered to have good follow-up compliance if they attend at least two follow-up visits; otherwise, they are considered to have poor follow-up compliance. Patient compliance will be assessed at weeks 1, 2, and 3.

In addition, we will also evaluate the participants’ application of the plaster (including application time, number of plasters applied, occurrence of plaster detachment, and medication experience), whether they used other treatments, and whether they engaged in vigorous exercise.

### Data collection and monitoring

#### Data collection

We will utilize an Electronic Data Capture (EDC) system for managing and collecting research data. All cases, whether they adhere to the trial protocol or are withdrawals, should be fully, accurately, and clearly documented in the CRF by the investigator based on the participants’ original observation records, in accordance with the trial protocol.

#### Data entry and modification

Data management will be handled by a data manager who is independent of this trial. The data manager will establish a database based on the trial protocol and the CRF, and configure logic verification procedures within the system. Logic verification can be conducted through both automatic system logic verification and manual logic verification. The data manager, in collaboration with the principal investigator, will develop data range checks and logic check contents according to the ranges and interrelationships of the various indicators in the CRF. Verification methods include system verification and manual medical verification. Before data entry, erroneous data entries will be controlled, the causes of errors will be identified and corrected, and all errors and modifications will be recorded and saved. After data entry, any corrections or modifications must be approved by the principal investigator at each center. All operations within the system are traceable.

### Statistical analysis

#### Statistical analysis data set

The participants used for statistical analysis in this trial will be classified and defined as follows:

- Full Analysis Set (FAS): This is defined as the collection of all cases that have been randomized, have used the study drug at least once, and have at least one set of efficacy evaluation data after drug administration. In case of missing primary outcome indicators, the last observation carried forward (LOCF) method will be used according to the Intention-to-treat (ITT) principle. The FAS is the primary analysis set. For secondary outcome indicators, only the FAS without LOCF will be used for analysis.
- Per-Protocol Set (PPS): This refers to the collection of cases that meet the inclusion criteria, do not meet the exclusion criteria, and have completed the treatment protocol. It includes cases that adhere to the trial protocol, have good compliance, and have completed the CRF as specified (PP analysis). The PP analysis is primarily used for the primary outcome indicator.
- Safety Set (SS): This is defined as the actual data from participants who have received at least one treatment and have safety outcome indicator records. Missing safety values will not be carried forward. The incidence of adverse reactions will be calculated using the number of cases in the safety set as the denominator.

#### Statistical analysis methods

We will use IBM SPSS Statistics (version 26.0, IBM, Armonk, New York) for data analysis. The significance level is set at 0.05. Non-inferiority is established if the upper limit of the 95% confidence interval for the difference between the two groups is below the non-inferiority margin.

For quantitative data such as WOMAC scores (pain, stiffness, and joint function dimensions), EQ-5D-5L scale assessments (converted to utility scores using utility values), and knee circumference and range of motion, the following statistical methods will be used based on whether the data are normally distributed: (1) If the data are normally distributed, statistical descriptions will include mean, standard deviation, minimum, and maximum values, and differences between the experimental and control groups will be calculated using independent-samples *t*-tests. (2) If the data are skewed, the median will be used to represent the average level, and the interquartile range will represent the degree of dispersion. Differences between the experimental and control groups will be calculated using the Wilcoxon-Mann–Whitney test.

For categorical data such as EQ-5D-5L scale assessments (frequency of each level in each dimension), severity of local skin reactions to the plaster (including subjective symptoms and skin reaction evaluations), rescue therapy usage rates, and adverse event incidence rates, frequencies and percentages will be used for descriptions, and differences between the two groups will be calculated using chi-square tests. For indicators with observations at different time points, repeated measurement ANOVA will be used to analyze the changes in the observed indicators over time.

## Discussion

This randomized, active-controlled, parallel-group, non-inferiority, multicenter clinical trial will evaluate the effectiveness and safety of PRP in participants with KOA. The trial plans to enroll 440 participants, allocated in a 1:1 ratio to the PRP group and the flurbiprofen Cataplasms group. All participants will receive one week of treatment, followed by a two-week post-treatment follow-up. We will assess participants’ pain using the pain dimension score of the WOMAC scale, and evaluate knee joint function using the stiffness and joint function dimension scores of the WOMAC scale (subjective indicators) and knee joint range of motion (objective indicator). We will assess joint swelling by measuring knee circumference. The improvement in participants’ quality of life will be evaluated using the EQ-5D-5L scale, with utility scores calculated based on utility values and changes in the frequency of each level across dimensions. For safety assessment, the trial will include blood laboratory tests, detailed recording of adverse events, and assessment of the severity of local skin reactions to the plaster based on a four-point Likert scale. Additionally, we will record information related to plaster application, patient compliance, and the use of rescue therapy. Before the study begins, we will provide consistency training to investigators at seven centers to standardize enrollment and assessment methods.

The head-to-head active-controlled non-inferiority design was selected based on the following rationale: (1) The clinical practice guidelines issued by the China Association for Chinese Medicine strongly recommend Chinese herbal medicated plaster as a topical therapeutic regimen for KOA patients (Level of evidence: I) (28); (2) flurbiprofen Cataplasms, as a topical NSAID formulation, has been widely used in clinical practice for managing swelling and pain in osteoarthritis, with its effectiveness and safety validated by multiple clinical trials (29–31). Topical NSAIDs are recommended as non-surgical treatment options in various clinical guidelines (1, 9); (3) Direct comparison with the current clinical standard topical therapy aligns with real-world clinical practice needs and provides physicians with direct comparative effectiveness evidence; (4) The use of an active comparator avoids exposing trial participants to ineffective treatments; (5) The non-inferiority study design allows verification of the investigational drug’s effectiveness and safety by demonstrating non-inferiority in relevant outcome measures when compared to a control agent with established effectiveness and safety profiles.

This trial has certain limitations. Due to the PRP’s dosage form, odor, and application method, developing a placebo control appears impractical. This limitation prevents participant and researcher blinding, creating potential performance bias through treatment awareness. However, the statistical analysis for this trial will be conducted by personnel unrelated to the trial, which can effectively avoid detection bias. The outcome measures observed in this trial, including the WOMAC score, EQ-5D-5L scale, and Severity of local skin reaction after application, all rely on the subjective judgments of participants, which may introduce bias. This trial incorporates the observation of objective outcome measures, which can effectively mitigate the bias introduced by participants’ subjective factors when assessing joint function and swelling.

In summary, this trial aims to investigate and explore the effectiveness and safety of PRP in the treatment of KOA, generating methodologically rigorous evidence for clinical decision-making. The findings will enhance medical understanding of PRP therapy, flurbiprofen cataplasms, and KOA, with potential translational benefits for future patients presenting similar clinical characteristics.

## Footnotes

^1^ www.ncss.com
